# On the Treatment of Neuralgia

**Published:** 1883-09

**Authors:** 

**Affiliations:** Member of the Academy of Medicine, Physician to the Hopital St. Antoine, etc., Paris, France


ON THE TREATMENT OF NEURALGIA. A Clinical Lecture by PROF. DUJARDIN-BEAUMETZ, Member of the Academy of Medicine, Physician to the Hopital St. Antoine, etc., Paris, France.

In speaking of the etiology and treatment of neuralgia, Prof. Dujardin-Beaumetz speaks of this disease as being the result of modifications affecting the integrity of the nervous system, central or peripheral, or of the blood, or finally of the circulation. Here, then, are three classes of causes which we must pass in review.

From the standpoint of the nervous system, leaving one side alterations, more or less profound, and compressions of the nerves, I desire to call your attention to two points: first, the predisposition to neuralgias in the neuropathic and hysterical; second, to inflammation of the nerve-tubes or neuritis.

Neuralgia is one of the manifestations of the nervous temperament, and it may be affirmed that every nervous person suffers more or less from it. In these cases we witness the triumph of the bromides and of hydrotherapy. Bromide of potassium (alone or associated with other bromides), cold douches, local or general, and suitable out-door exercise are the most certain methods of cure of neuralgias due to neuropathy; electricity, and especially static electricity, is a useful adjunct.

Neuritis is one of the causes of obstinate neuralgias; it entails atrophy of the member, and trophic cutaneous disturbances. The most effective remedy for neuritis is revulsion, which has a favorable influence on both pain and inflammation. Here all the revulsive methods before alluded to are applicable, and especially active cauterizations. To counteract the trophic troubles, you should have recourse to galvanism.

Neuralgias due to circulatory modifications are of two kinds—congestive and anæmic. Congestive neuralgias are especially frequent in the arthritic, and are best treated by ergot and aconitia. It is especially in facial neuralgias of plethoric persons, with marked congestion of the face, that aconitia is effective, and this by a sort of double influence on the circulation and on sensory innervation. Marino* has recently proposed ergotine for these congestive neuralgias, basing his recommendatien on the known effects of this drug in constricting the minute vessels and anæmiating the tissues. I have never used this drug in neuralgia, having almost always found in aconitia a heroic and certain remedy. In neuralgias from cerebral anæmia, morphia is *par excellence* the medicament. It removes pain and provokes a salutary congestion of the sensory nerve-centers, which directly antagonizes the cause of the pain. Gymnastic exercises and hydrotherapy are most excellent adjuvants in these cases.

---

* Marino, Ergotina per]use Epidermico, nelle cure delle Neuralgia, Palermo, 1877.

As for the alterations of the blood, which are the point of departure of dyscrasic neuralgias, they are very numerous, and chlorosis deserves the first mention. It may be affirmed that every chlorotic girl is neuralgic, and here we have an illustration of that grand principle of Hippocrates, *sanguis moderator nervorum.* Therefore, every therapeutic means which shall augment the richness of the blood, and the proportion of its hæmoglobin, is appliable to these cases; ferruginous or arsenical medication, baths of compressed air, inhalations of oxygen, country air and exercise, hydrotherapy, gymnastics, nourishing food, all these remedial measures are indicated. I cannot too strongly urge the use of arsenic in these cases of chlorosis; in my experience, the arsenical treatment has been quite as successful as the ferruginous, if not even more so; for arsenic acts, not only as a reconstituent, but it is a direct modifier of nerve substance. Those who have had the most practical acquaintance with the use of arsenic in neuralgia are the most emphatic in its praise.

This medicament may be given in the form of Fowler's solution; dose, three to ten drops after meals. [Anstie prefers this form of administration; he finds arsenic especially useful in neuroses of the parvagum.] It is also given in granules and in pill form. [The combination with quinine, ext. aconite, and strychnine of Dr. Gross, known as Gross's neuralgic pill, is a favorite one in the United States.]*

Certain neuralgias are of malarial origin; here we witness the triumph of sulphate of quinine. Marrotte has given us a good description of these febrile neuralgias, due to marsh poison. Where there is any reason to suspect any pathogenetic influence of this kind, you should be particular to ascertain whether the neuralgic attack comes on at a fixed hour, in which event you can easily and speedily control the affection by a full dose of quinine. You can increase the effect of the quinine by combining it with aconite, giving every three hours a capsule or wafer containing one-quarter of a milligramme of crystallized nitrate of aconitia, and twenty-five centigrammes of quinine; four of these may be given each day.

---

**Vide* Cohen on "Arsenical Treatment of Neuralgia" in *Journ. Med. de Bruxelles*, 1864. Barilla, in do., for 1863. Vanlair, The Neuralgiæ, their Forms and Treatment, etc., 1882.

The diatheses, and in particular, syphilis, arthritism, dartre, have often a marked etiological relation to neuralgia. As for the first, you must not confound the so-called "osteoscopic pains" with the neuralgic pains, which really often exist under the influence of syphilis. These cases demand the ordinary anti-syphilitic treatment.

As for neuralgias of arthritic origin, they are among the most frequent, and sciatica and gout are often one and the same thing. The means which succeed the best in the treatment of gouty and rheumatic neuralgias are baths, and in particular, sulphur baths and vapor baths; the latter are often medicated to advantage with turpentine or pine shavings. In rheumatic neuralgias, the thermal waters are often successful; those of Plombières, Bourbonne, and especially Aix-les-Bains. In these neuralgias we may employ cyanide of zinc, proposed by Luton, and especially salicylate of sodium.

The dartrous neuralgias are readily amenable to the arsenical treatment.*

Such is a somewhat curt summary of the principal indications of the pathogenetic treatment of neuralgia. I must now briefly consider the therapeutics of certain forms of neuralgia, and for convenience of arrangement will begin with the foot and end with the head.

Plantar neuralgia is one of the most painful of neuralgias, and incapacitates the suffer from walking or standing. It is especially gouty and rheumatic patients that are affected in this way. This neuralgia is often rebellious, lasting months, and even years. You have seen a good example in our wards; I allude to a certain female who, in consequenee of rheumatism following an accouchement, has been for six months confined to her bed from plantar neuralgia. What has seemed to succeed best in her case has been the application of strong tincture of iodine, and hot sulphur foot-baths.

I shall not dwell long on sciatic neuralgia, which has been

---

*Marrotte. "Febri-neuralgiés de l'esthime du Gosier," *Bull. Gen. de Therap.*, 1874. Abbot "Sciatic and Facial Neuralgia Treated by Salicylic Acid and Salicylate of Sodium," *Boston Med and Surg. Journ.*, July, 1879. E. Labbé, "Neuralgiés traités par le Salicylate de Soude," *Soc. de Therap.*, Paris, Feb. 9, 1881. Lagrelette, "De la Sciatique," *Th. de Paris*, 1869. This writer advocates strongly vapor baths and hydrotherapy in sciatica.

often taken as the type of neuralgia. Here the revulsive medication, carried out in all its rigor, succeeds best. Sciatica is often a neuritis, and it may almost with certainty be affirmed, when this neuralgia is obstinate, and is not due to compression of blood-vessels, viscera, etc., that it proceeds from inflammation of the nerve. I believe that this frequency of neuritis of the sciatic nerve results from proximity of that nerve to the surface, and from the modifications which it is likely to experience from external influences, and especially from atmospheric changes; there is certainly no form of rheumatic neuralgia so common.

*Apropos* of these stubborn sciatic neuralgias, I must remark that they are often dependent on affections of the spine, especially when they are bilateral. Essential double sciaticas are very rare, and when they occur are generally occasioned by tabes dorsalis, or, as Worms has shown, by diabetes.*

Neuralgia of the uterus, bladder, testicle, and spermatic cord have frequently been observed. I know that it has been disputed whether these *visceralgias* ought to be considered true neuralgias, but it is of little consequence what we call them, they are painful affections, and prompt relief is demanded. In uterine neuralgia, cauterizations are of striking utility. You will, in fact, observe a certain number of females, who, apart from all organic disease of the uterus, suffer pains in that organ presenting all the distinctive characters of neuralgia. In these cases revulsive applications to the os or cervix with the Paquelin cautery, or the acid nitrate of mercury, give excellent results. Do not, however, forget that in this neuralgia of the organs contained in the pelvis, one of the best methods of administering anodynes is the suppository. [Here the morphia suppository of the U. S. P., with one-third grain extract of belladonna, will do good service.]

Ileo-lumbar neuralgia is often the cause of cruel suffering, besides being rebellious to the most energetic treatment. In fact, this affection is frequently due to profound troubles of the kidney, and particularly to renal lithiasis. You are aware that in these renal cases of persistent neuralgia it has been proposed to remove, or to open, the kidney; in a word, to perform nephrectomy or nephrotomy, as Professors Leon Le Fort and Le

* Worms, Symmetrical Sciaticas in Diabetes, Paris, 1880.

Dentu have done. I pass by gastralgia, hepatalgia, and the greater part of abdominal neuralgias, only referring you to what I have already said in regard to them while treating of diseases of the stomach, liver, and intestines, and I come now to intercostal neuralgia. This is a very common neuralgia, and all delicate, nervous women suffer from it more or less. Peter, in his remarkable lessons on *pains in the side*, insists that intercostal neuralgia is always limited to the left side; I do not quite agree with him in this. It is true that the far greater part of painful intercostal affections are on the left side. You will nevertheless now and then see hysterical patients whose painful sensations and whose anæsthesia are exclusively right-sided. On whichever side it may occur, this neuralgia is obstinate, and resists not only morphine injections, but also revulsive treatment. Hydrotherapy, applied in the form of douches to the painful region, seems to me one of the best means of combating this rebellious intercostal neuralgia.

I shall finish this lecture by a brief consideration of the neuralgias of the fifth nerve.

Odontalgia, I need not tell you, is one of the most common of painful affections, and every one has at some time experienced the atrocious pain of toothache. This neuralgia is often determined by alveo-periostitis, or by a carious tooth, which affects the terminal portion of the dental nerve. There exists a ready means of relief for this pain in arsenious acid, which destroys the dental pulp, a method which Tomes, Magitot, and Combe have advised. A paste is recommended (to be applied on cotton to the cavity of the tooth), consisting of two parts of white arsenic, two of morphia, one of gum tragacanth, and one of glycerine. Among the numerous measures which have been employed against odontalgia, Bouchaud has counselled electricity. His method is to place the positive pole over the diseased nerve, and the negative pole a short distance from it, and to pass a mild continued current.

A word now about facial neuralgia, properly so called. These neuralgias affect sometimes the supra-orbital nerve, sometimes the infra-orbital branches; these last are the most obstinate. As I have already told you, they often yield readily to treatment by aconitina, or to sulphate of quinine when of an intermittent character. They sometimes defy all treatment, and have been known to involve the facial nerve, as well as the fifth. Without discussing the question of recurrent neuralgia—a subject which has of late been treated in a brilliant manner by Cartaz—you all know that neuralgia is often accompanied by painful contractions, and that to this syndrome the name has been applied of epileptiform neuralgia, or tic douloureux of the face. It is the most atrociously painful affection that has ever afflicted humanity, and instances have been known where it has driven its unhappy victim to suicide.

It is in these cases that surgery steps in, with its nerve-stretching and neurectomy; here, too, the advantages of galvanism have been lauded. If you employ electricity you must never exceed a certain intensity (two or three milliampères, for instance); you must also, as Apostoli enjoins, make use of rheostats, and interpose a certain resistance to the current, to avoid the *photopsias* which are produced with each modification of the current. It is understood that the positive pole must be placed over the painful point, and as for the duration of the *sèance*, it ought to be continued till the pain disappears.

I must, before finishing, say something about migraine, which (therapeutically, at least) belongs to the neuralgias. You are not ignorant of the discussions which have arisen over the pathogeny of migraine, some considering it simply a neuralgia of the trifacial, others as a special neurosis of the same nerve, or even as a neuralgia of the brain itself, a cerebralgia, as Romberg maintains. There are still others who assert, as does Du Bois-Reymond, that the principal seat of migraine is the cervical portion of the great sympathetic. This is the view generally held in France, especially by Gubler and Jaccoud. It is, in fact, probable that migraine is not a simple neuralgia, but a complex neurosis, affecting alike the cerebrum, the trigeminal nerve, and the cervical portion of the sympathetic.*

Whatever its pathological nature, migraine is a very distress-

* Tissot, *Nerves and their Diseases*, t. xi , Paris, 1873. Bouillaud, *Nosographie Medicale*. Pelletan, *Coup. d'œil sur la Migraine et sur ses divers traitements*. Du Bois-Reymond, *Archiv. fur Anat.*, 4th livra:son, 1860, p. 461. Gubler, *Dict. des Sc. Med.*, Art "Migraine." Piorry, *Memoire sur la Migraine, Traite, Med. Pratique*, t. viii. p. 73.

ing affection, and you will often be consulted in reference to it. You ought always, if possible, to ascertain the first cause, and as far as this is concerned, this is what you will discover: in nine cases out of ten migraine is a diathetic affection, and for my part I have frequently observed it in hæmorrhoidal, arthritic, and asthmatic subjects. Your treatment then should be directed to the arthritic diathesis, of which the migraine is an expression. At other times you will find the migraine due to causes of a different character, occasional causes to which you should address your therapeutic endeavors. These causes may be ranged in three groups; first, excess of work, and especially brain work during the night-time, and with the aid of too strong a light. Piorry referred all migraines to fatigue of the the eyes; to him migraine was only a manifestation of irisalgia. Second, anæmia; the megrim of chlorotics is an example, coming on whenever by any cause the organism is enfeebled. Third, congestive head-troubles, instances of which we see in the hemicranias of the gouty or arthritie.

The first class of patients are benefited by rest from mental toil, and by bromide of potassium; the second may require hydrotherapy and morphia; the third will need alkalies, intestinal derivatives, and especially aconitia.

I have finished this long exposition of the treatment of neuralgia—exposition far from complete, notwithstanding its length. I believe, however, that I have furnished you the general principles which ought to guide you in your practice.

In the combat with pain your therapeutic resources will be taxed to the uttermost, and you can only fulfil your professional duty by the intelligent endeavor *always to relieve, if you cannot cure.—Medical News.*

